# Impact of pulmonary valve replacement on left and right ventricular function using strain analysis, in children with repaired tetralogy of Fallot

**DOI:** 10.1186/s43044-023-00379-w

**Published:** 2023-06-19

**Authors:** Maryam Moradian, Fariba Rashidighader, Fatemeh Golchinnaghash, Mahmoud Meraji, Hamid Reza Ghaemi

**Affiliations:** Department of Pediatric Cardiology, Rajaei Cardiovascular Research and Medical Center, School of Medicine, Iran University of Medical Sciences, Intersection of Niayesh Highway and Valiasr St, Tehran, 1995614331 Iran

**Keywords:** Echocardiography, Tetralogy of Fallot, Pulmonary valve incompetence

## Abstract

**Background:**

In repaired Tetralogy of Fallot (rTOF), pulmonary regurgitation and resulting right ventricular (RV) and left ventricular (LV) dysfunction are associated with adverse clinical outcomes. We performed an echocardiographic assessment of LV and RV function using Global Longitudinal Strain (GLS) and conventional echo method prior to and following Pulmonary Valvular Replacement (PVR) to help inform proper timing of operation.

**Results:**

A total of 30 rTOF patients (12.17 ± 2.5 years, 70% male) were included. Regarding to LV function, the study revealed a significant reverse correlation between LV GLS (absolute value) and early (mean = 10.4 days) and late (mean = 7.4 months) postop LVEF. Paired T-Test showed significant difference between GLS of LV and RV before and late after operation (op), however, without significant changes early postop. Late postop significant improvements occurred in other conventional echo indices of LV and RV function as well. There was also a significant correlation between echo-measured LVEF & Fraction Area Change (RV FAC) and MRI-derived LVEF & RVEF, respectively.

**Conclusion:**

In this cross-sectional study in rTOF patients, RV and LV GLS as well as conventional echocardiographic indices regarding LV and RV function improved significantly after 6 months (mean = 7.4mo) following PVR.

## Background

Primary repair of TOF at early childhood has low mortality, but some patients still remain with long-term morbidity in adulthood because of complications such as PR leading to progressive RV dysfunction and ultimately RV failure, electromechanical dissociation, recurrent RV outflow tract obstruction, other residual defects, arrhythmias, sudden death (SD) and so on [1–4]. RV dimension and function are notably affected by corrective surgery of TOF; however, LV dysfunction, explained by adverse ventricular interdependence, arrhythmia and late-onset complete heart block (CHB) could also be other important risk factors for unfavorable clinical outcome and worrisome occurrence of late SD [5–9].

To overcome these comorbidities, to mitigate deteriorating effects of long-term exposure to RV volume overload, and subsequently improve clinical outcome of rTOF survivors over time, some patients are selected for PVR based on available guidelines, when decreasing in exercise tolerance, deteriorating RV size and function, occurrence of significant arrhythmias and so on [8, 10]. Suitable timing of PVR is of decisive importance, as instant RV remodeling (during the first postop years) with reduction in RV volume and mass has been observed after early PVR [4, 11–13]. However, it should be noted that reverse RV remodeling does not occur in every patient and adverse events still occur frequently after PVR because, based on evidence, compensatory mechanisms of the RV myocardium ultimately fail and dysfunction might be irreversible [4, 14]. Despite a large number of studies, controversies on the necessity, surgical techniques and optimal timing of PVR have ever being remained, although it has been held that normalization could be achieved in 70% of patients as long as preoperative RV end-diastolic volume (RVEDV) < 159 mL/m^2^ or End-systolic volume (RVESV) < 82 mL/m^2^ is not slipped by [15–18].

A better and an earlier understanding of cardiac function can help improve indications, optimal timing and subsequently outcome of PVR. Strain analysis by speckle tracking alongside of conventional echocardiography can elucidate the information required to the best determination of cardiac function. Speckle tracking-based strain is a newer index of ventricular performance that measures local myocardial deformation; moreover, it may be more sensitive indicator than EF [18–22]. RV GLS and LV circumferential strain (LV GCS) absolute values both have been shown to inversely correlated with RV size [18–20]. Accounting for newer techniques such as 3D and deformation study, echocardiography retains a key role in following up of ventricular function, although to precisely validate the information, CMR is sometimes needed [21, 22]. It should be emphasized that performing MRI to inform accurate timing is neither feasible, particularly in pediatrics, nor accessible adequately and nor cost effective and sometimes, it is impossible due to pacemakers, Intra-Cardiac Defibrillator (ICD) and mechanical prosthetic valve possibly implanted. Given that it is still currently debatable how to determine the optimal time frame for PVR [4] and because of abovementioned drawbacks concerning MRI, we designed this study to carry out echocardiography, considering deformation and conventional analysis, as an investigation to compare RV and LV function prior to and following the operation of PVR. It is hoped that by making more evidence in the field of pediatrics, we could take a step in improving outcome of these patients, as the small sample size, paucity of studies and hard outcomes have limited the experience in this age group.

## Methods

### Study design

This was a prospective cross-sectional study on rTOF population undergoing a prosthetic PVR, designed to evaluate changes in deformation and conventional echocardiographic indices before and after PVR. We implemented the study in pediatric age group, all of which were candidate of PVR following rTOF during the time of study (about 8mo) due to severe PR, enlargement and dysfunction of RV, also with suitable echo images for speckle tracking analysis. Exclusion criteria included severe RV outflow obstruction and residual Ventricular Septal Defect (VSD). All patients appraised by MRI before PVR but 4 patients, for which MRI could not be done due to existence of pacemaker or other accountable problems. Total of echocardiographic indices were measured before PVR and also, early and late follow-up periods after the operation.

On echocardiography, LV function was assessed by LVEF (by M mode and biplane Simpson methods), LVOT VTI (LV Outflow Tract Velocity Time Integral) and LV GLS; RV function estimated by TAPSE (Tricuspid Annular Plane Systolic Excursion), Sprime (peak systolic velocity by tissue Doppler, RV Sm), FAC and RV GLS. Echo deformation study included using LV and RV GLS was done by an expert pediatric cardiologist and later rechecked by the same Dr and then, acquisited features reanalyzed by a Pediatric cardiology fellow again. The study protocol included a pre-operative, an early (7–14 days) and a late (6–9 months) postoperation echocardiographic assay. A Philips iE33 xMATRIX echocardiographic system with a QLAB quantification using a S 1–5 MHZ Cardiac Probe was used for taking images. Acceptable standard views from 2-chamber, 3 chamber & apical 4-chamber with a superimposed ECG (lead 2) were used for measurements. LVOT VTI was obtained using PW Doppler technique at a sweep speed of 50–100 mm/s, so were TAPSE from M Mode view, FAC from 2D and SM RV by DTI study.

Average RV and LV myocardial GLS were assessed with speckle tracking echocardiography technique on those acquisited optimal echo views. CMR would be performed at interval of less than 6 months from preoperation echocardiography in which LVEF and RVEF were determined.

### Statistical analysis

All data were evaluated with SPSS 26 where descriptive study was done for qualitative and quantitative variables; Spearman and Pearson coefficients were used for assessment of correlation between them. Normality assumption was determined using Kolmogorov–Smirnov and Shapiro–Wilk tests. Difference between patients before PVR and early and late after that was appraised with Paired T test by repeated measures. Then, for determination of similarity between MRI-derived and echo-derived LVEF and RVEF, firstly one sample T test was used to determine how significant the difference is, next measuring mean value of those two variables and finally, the difference and mean values assessed by Bland–Altman plot using accounting 2 SD for upper and lower reference lines were illustrated. *P* values < 0.05 were considered significant. Interobserver and intraobserver variability were evaluated by agreement coefficient of kappa.

### Ethical statement

Written informed consents were taken from patients or their parents whether to participate in the research, explaining that the subjects’ confidentiality was maintained, associated with ethical code of (IR.IUMS.FMD.REC.1401.2.13) which determined by Iran University of Medical Science(IUMS) (on 3.5.2022), although performing echocardiography was based on their postoperative clinical follow-up programs, so it did not impose any extra financial and other troubles to them. It should be noted that our study does not report on or involve any animal data or tissue.

## Results

We included 30 patients (mean age12.17 ± 2.5 years, 9 female), all of which were candidate of PVR following rTOF due to enlargement and dysfunction of RV and severe PR. Demographic characteristics are listed in Table 1.

**Table 1:** Demographic characteristics of patients with PVR

Variables	Mean ± SD, percent
Age (year)	12.17 ± 2.5
Age of operation (year)	2.86 ± 1.88
Surface area (m^2^)	1.29 ± 0.28
Gender (male/female)	2.33 (70%male)
Symptoms	
No symptom	3 (10%)
Dyspnea	23 (76%)
Dyspnea + fatigue	4 (13%)
Type of valve	
Biologic	22 (73%)
Mechanical	7 (23%)

There was no paravalvular leakage, acceptable hemodynamic study, and no significant dysrhythmia in all of the patients who were normally distributed according to Kolmogorov–Smirnov test, excluded one patient whom operation delayed due to symptomatic coronavirus. Also, there was no mortality during a total of 6–9-month follow-up period. Pearson and spearman coefficients showed no significant correlation between LVEF with patients’ gender and symptoms. However, it revealed a significant inverse correlation between patients’ age, age difference between rTOF and PVR and surface Area (SA) with LVEF, although there was no significant relationship between age and LV & RV GLS before and after PVR. However, there was also a nonsignificant inverse correlation between RVEF and age-related data (Table 2).

**Table 2:** Important correlations between pre-operation MRI, echocardiographic data and some demographic data

	RVEF MRI	LVEF MRI	LVEF echo	LV GLS	RV GLS	RV FAC	LVOT VTI	TAPSE	RV Sm	Age	Age–age sx	SA
RVEF MRI	1	*R* = − 0.035	*R* = 0.001	*R* = − 0.48	*R* = − 0.36	*R* = 0.94	*R* = 0.47	*R* = 0.24	*R* = 0.07	*R* = 0.2	*R* = − 0.15	*R* = − 0.1
		*P* = 0.86	*P* = 0.99	*P* = 0.011*	*P* = 0.05*	*P* = 0.000**	*P* = 0.02*	*P* = 0.22	*P* = 0.70	*P* = 0.32	*P* = 0.4	*P* = 0.62
LVEF MRI	*R* = − 0.035	1	*R* = 0.90	*R* = − 0.16	*R* = − 0.22	*R* = − 0.11	*R* = 0.06	*R* = 0.1	*R* = 0.42	*R* = − 0.58	*R* = − 0.41	*R* = − 0.49**
	*P* = 0.86		*P* = 0.000*	*P* = 0.43	*P* = 0.26	*P* = 0.58	*P* = 0.75	*P* = 0.65	*P* = 0.03*	*P* = 0.002**	*P* = 0.03*	*P* = 0.008
LVEF echo	*R* = 0.001	*R* = 0.90	1	*R* = − 0.41	*R* = − 0.38	*R* = 0.02	*R* = 0.19	*R* = − 0.1	*R* = 0.49	*R* = − 0.48	*R* = − 0.42	*R* = − 0.47**
	*P* = 0.99	*P* = 0.000*		*P* = 0.025*	*P* = 0.03*	*P* = 0.9	*P* = 0.3	*P* = 0.60	*P* = 0.006**	*P* = 0.007**	*P* = 0.02*	*P* = 0.009
LV GLS	*R* = − 0.48	*R* = − 0.32	*R* = − 0.41	1	*R* = 0.6	*R* = − 0.47	*R* = − 0.4	*R* = − 0.25	*R* = − 0.33	*R* = 0.18	*R* = 0.01	*R* = 0.102
	*P* = 0.011*	*P* = 0.05*	*P* = 0.025*		*P* = 0.000**	*P* = 0.008**	*P* = 0.03*	*P* = 0.17	*P* = 0.07	*P* = 0.34	*P* = 0.95	*P* = 0.59
RV GLS	*R* = − 0.36	*R* = − 0.22	*R* = − 0.38	*R* = 0.6	1	*R* = − 0.38	*R* = − 0.53	*R* = − 0.36	*R* = − 0.54	*R* = 0.08	*R* = 0.11	*R* = 0.108
	*P* = 0.05*	*P* = 0.26	*P* = 0.03*	*P* = 0.000**		*P* = 0.04*	*P* = 0.003**	*P* = 0.04*	*P* = 0.02*	*P* = 0.64	*P* = 0.57	*P* = 0.67
RV FAC	*R* = 0.94	*R* = − 0.11	*R* = 0.02	*R* = − 0.47	*R* = − 0.32	1	*R* = 0.4	*R* = 0.28	*R* = 0.11	*R* = 0.16	*R* = − 0.12	*R* = − 0.12
	*P* = 0.000**	*P* = 0.58	*P* = 0.9	*P* = 0.008**	*P* = 0.09		*P* = 0.03*	*P* = 0.12	*P* = 0.57	*P* = 0.93	*P* = 0.53	*P* = 0.51
Age	*R* = 0.2	*R* = − 0.58	*R* = 0.48	*R* = − 0.12	*R* = 0.08	*R* = 0.23	*R* = 0.16	*R* = 0.22	*R* = 0.16	1	*R* = 0.70	*R* = 0.80
	*P* = 0.32	*P* = 0.002**	*P* = 0.007**	*P* = 0.52	*P* = 0.64	*P* = 0.21	*P* = 0.4	*P* = 0.24	*P* = 0.39		*P* = 0.000**	*P* = 0.000**

Correlation is significant at 0.01 level**, Correlation is significant at 0.05 level*; Age–Age Sx = Age of patient minus age of surgery, *SA* Surface area, all abbreviations are noted through txt

**Fig. 1:**
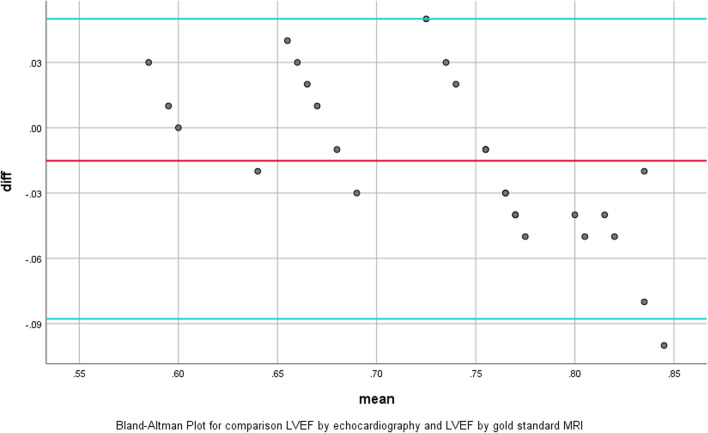
Accuracy of echo-derived LVEF based on CMR as gold standard. Bland–Altman plot for comparison of LVEF by echocardiography and CMR; mean difference between LVEF derived by echo and MRI = − 0.0152, SD = 0.37, *P* = 0.043

**Fig. 2:**
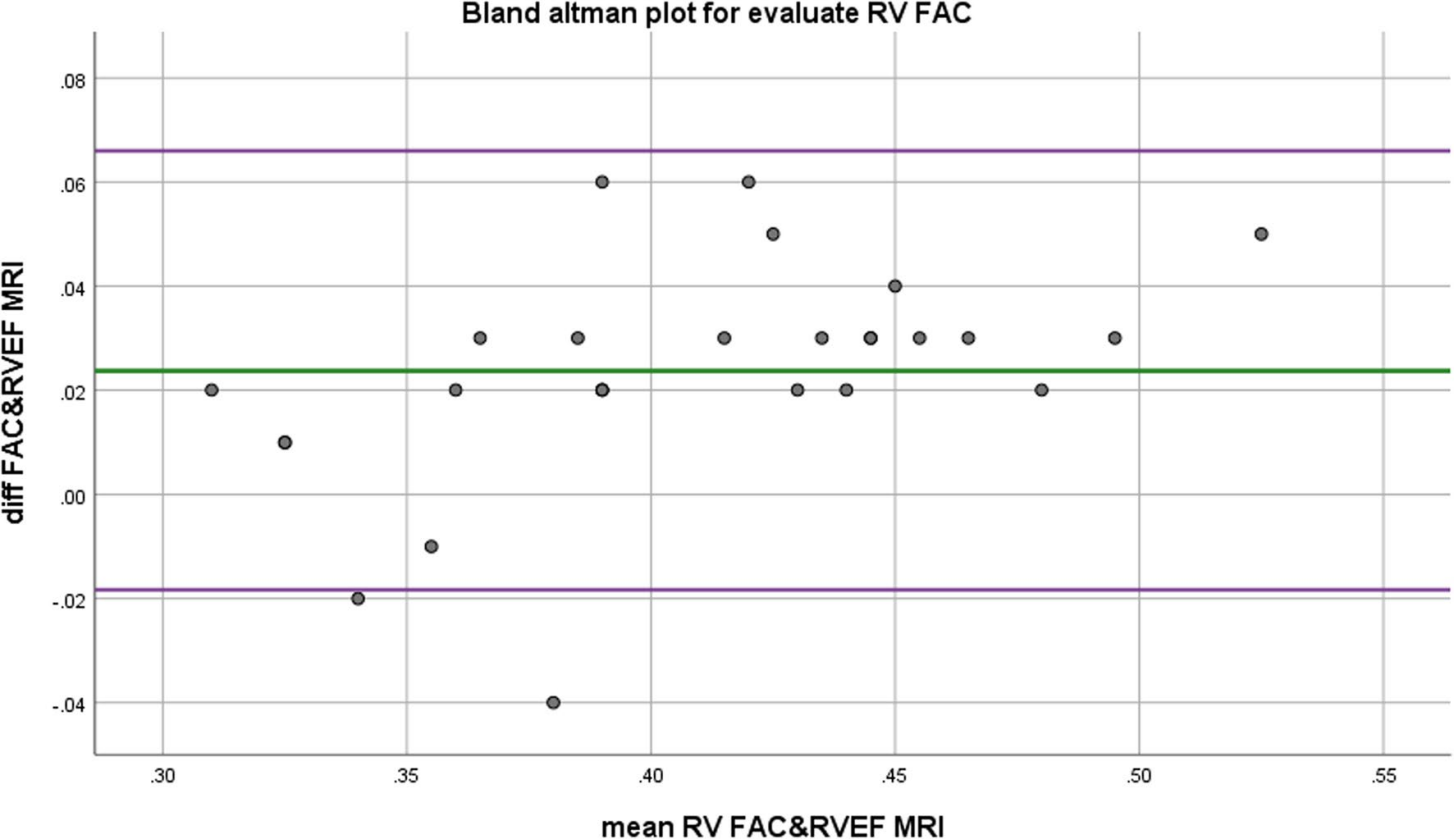
Accuracy of RV FAC by echocardiography based on CMR as gold standard. Bland–Altman plot for comparison of RVEF by echocardiography (RV FAC) and Cardiac MRI; mean difference between RVEF derived by echo and MRI = 0.0237, SD = 0.021, *P* = 0.000

Quantitative echocardiographic data were evaluated in patients prior to and following PVR at early and late post-operation where the early period was about 7–14 days (mean = 10.6 days), and the late study was performed during a 6–9 month (mean = 7.4mo)-follow-up period. Considering to RV function, there was significant inverse correlation between RV GLS with RV Sm, TAPSE and FAC. There were also significant relationships between RVGLS and LVGLS with LVEF and RV FAC, respectively (Table 2). Moreover, regarding to LV function, it was revealed a significant inverse correlation between LV GLS with early postop LVOT VTI, LVEF and late postop LVEF. MRI was not done for four patients before operation due to pacemaker implantation; in addition, postop MRI was not done for none of the patients due to mechanical valve for most of them, and acceptable results following operations. Conversely, a significant correlation was seen between echo-measured LVEF & RVEF and MRI-obtained LVEF & RVEF, respectively, which have been shown on Bland–Altman Plot (Figs. 1 and 2).

**Fig. 3:**
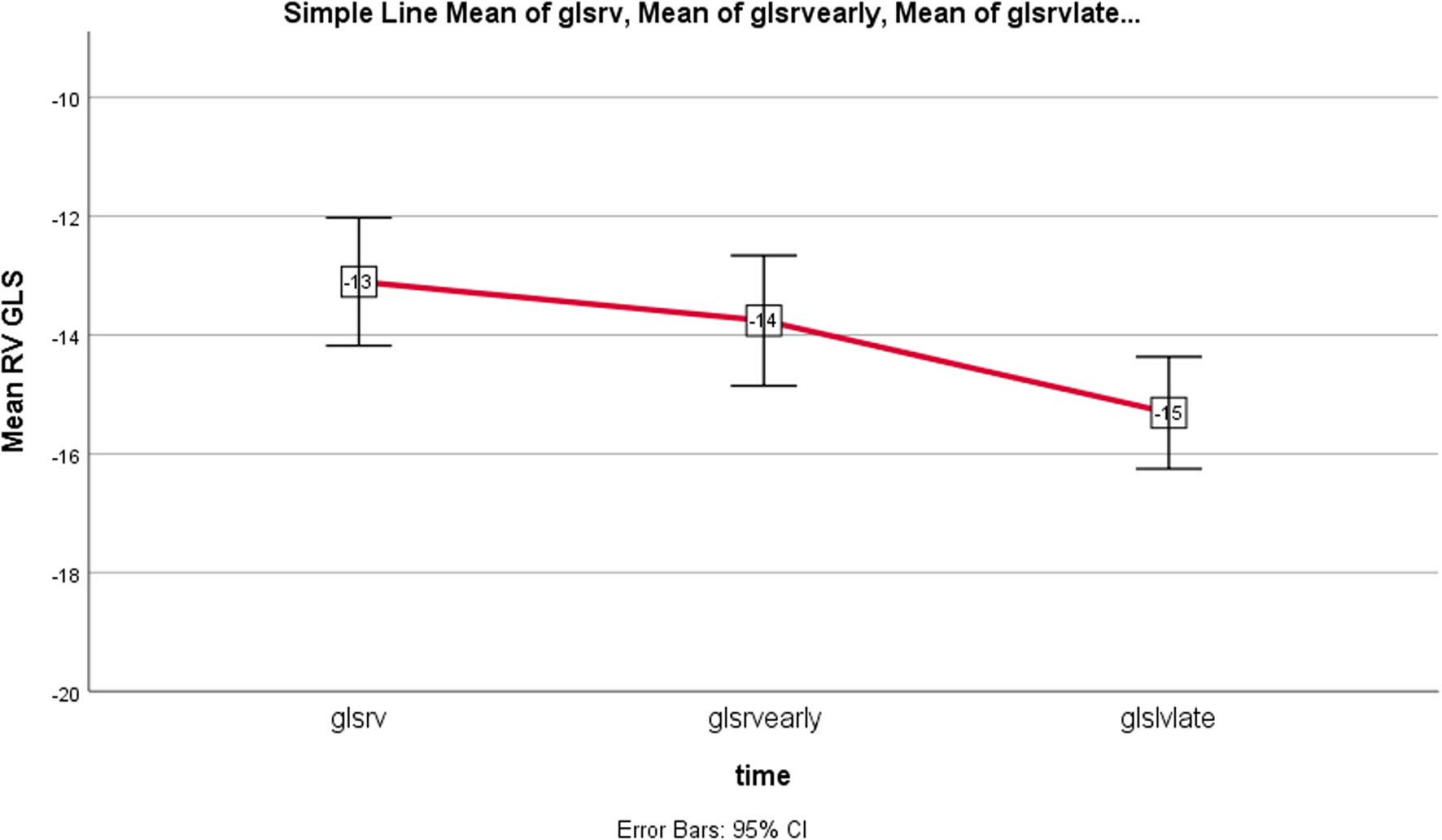
Downward slope of RV GLS changes over time; before, early and late after PVR, Baseline to early changes: *P* = 0.052, Baseline to late and early to late changes: *P* = 0.000: significant *P* < 0.05

**Fig. 4:**
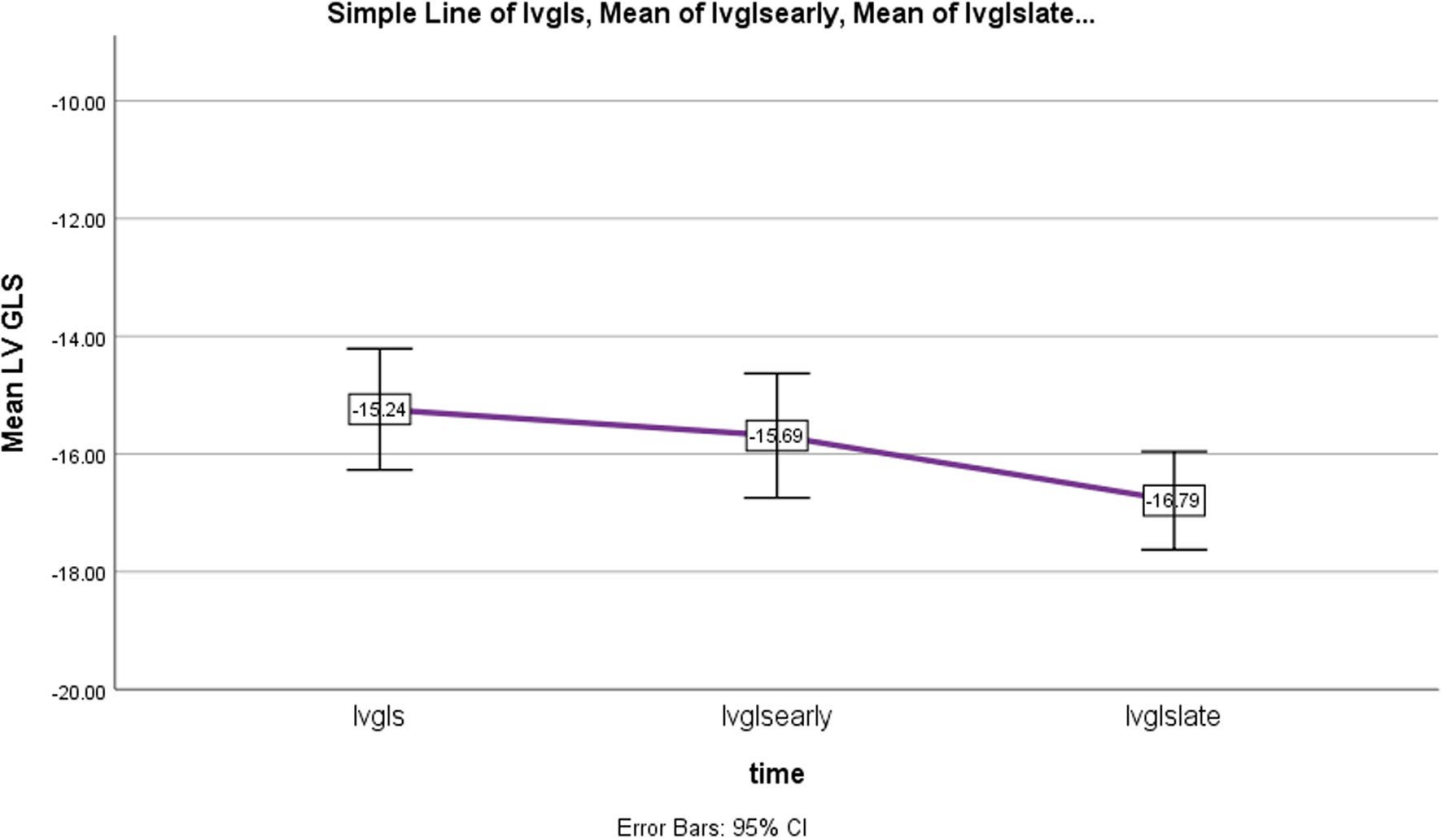
Downward slope of LV GLS changes over time; before, early and late after PVR, Baseline to early changes: *P* = 0.27, baseline to late: *P* = 0.000, Early to late: *P* = 0.005,: significant *P* < 0.05

**Fig. 5:**
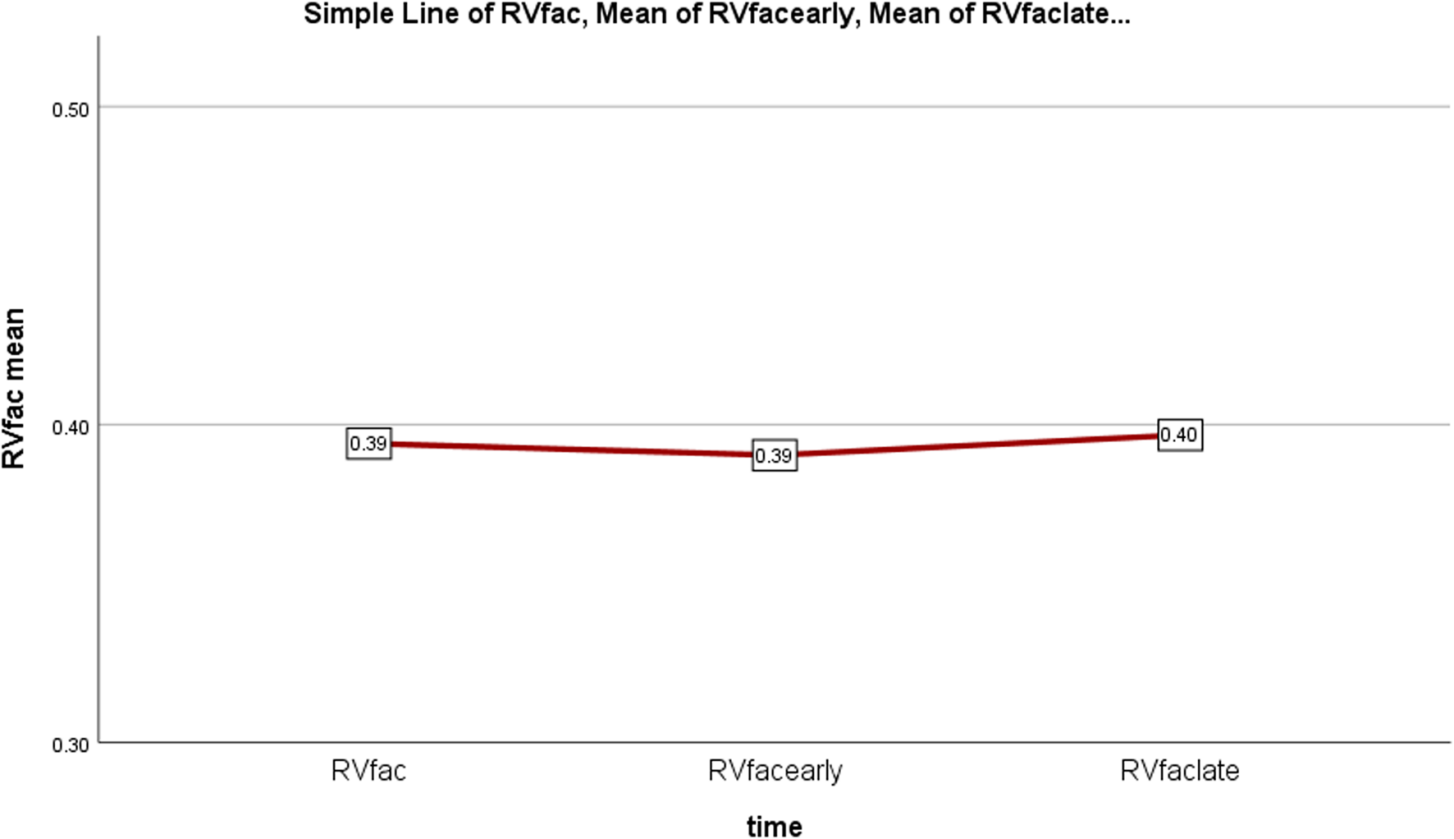
Upward slope of RV FAC changes over time; before, early and late after PVR. Baseline to early postop changes was not significant: *P* = 0.782, Late relative to early postop was significant: *P* = 0.001, late postop compared to baseline was not significant: *P* = 0.002

**Fig. 6:**
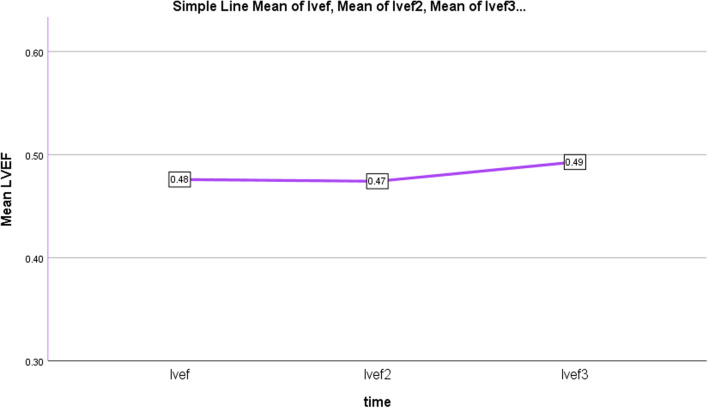
Upward slope of LVEF changes over time; before, early and late after PVR. Late postop relative to early postop was significant: *P* = 0.007, but late postop compared to baseline and baseline to early postop changes were not significant: *P* = 0.4; lvef2: early LVEF, lvef3: late LVEF

Regarding to the obtained results comparing patients pre and post-PVR, Paired T-Test showed significant difference between GLS of LV and RV before and late post-op, however, without significant changes early after operation (Table 3), as illustrated in Figs. 3 and 4. Late postop significant improvements occurred in other conventional echo indices of LV and RV function as well (Table 3, Figs. 5 and 6). Intraobserver and interobserver variability revealed a significant agreement of kappa correlation (Table 4).

**Table 3:** The results of ventricular function prior to and following PVR

Variables	Unit	Preop, mean ± SD	Early postop, mean ± SD *P* value	Late postop, mean ± SD *P* value
GLS LV	% Percent	− 15.24 ± 2.7	− 15.69 ± 2.77	− 16.79 ± 2.19
			*P* = 0.267	*P* = 0.000
GLS RV	% Percent	− 13.10 ± 2.83	− 13.76 ± 2.88	− 15.31 ± 2.48
			*P* = 0.052	*P* = 0.000
RV Sm	Cm/s	7.32 ± 1.16	7.31 ± 1.2	8.05 ± 1
			*P* = 0.932	*P* = 0.000
TAPSE	cm	1.33 ± 0.24	1.29 ± 0.27	1.50 ± 0.24
			*P* = 0.318	*P* = 0.000
LVOT VTI	cm	16 ± 1.79	16.12 ± 1.94	17 ± 1.8
			*P* = 0.684	*P* = 0.005
LVEF echo	% Percent	%47.6 ± 0.046	%47.4 ± 0.041	%49.3 ± 0.029
			*P* = 0.782	*P* = 0.002
FAC echo	% Percent	%39.4 ± 0.04	%39 ± 0.05	%39.6 ± 0.05
			*P* = 0.2	*P* = 0.4 (early & late)
				*P* = 0.007 (pre & late)
LVEF MRI	% Percent	%49 ± 0.07		
RVEF MRI	% Percent	%42 ± 0.06		
RV ESV MRI	ml	93 ± 29.5		
RV EDV MRI	ml	160 ± 41		

The results with statistical significance of LV and RV function indices prior to and following PVR, *RV EDV* RV end diastolic volume, *RV ESV* RV end systolic volume

**Table 4:** Inter- and intraobserver measurements of LV and RV GLS

	Intraobserver variability agreement (kappa)	Intraobserver variability (*P* value)	Interobserver variability agreement (kappa)	Interobserver variability (*P* value)
GLS RV preop	0.295	000	0.346	000
GLS RV early postop	0.309	000	0.293	000
GLS RV late postop	0.346	000	0.381	000
GLS LV preop	0.194	004	0.385	000
GLS LV early postop	0.225	000	0.565	000
GLS LV late postop	0.198	000	0.284	000

Inter- and intraobserver measurements of LV and RV GLS agreement with their significance (based on kappa coefficient)

## Discussion

Our data demonstrate that the majority of improvement in RV and LV function after surgical PVR took place at more than 6 months after operation, although a modest recovery is also occurred within days (Figs. 3, 4, 5 and 6, Table 3). The reason lies in reduction in RV volume overload which in turn affects LV function based on positive ventricular interdependence theory and disturbing effect of enlarged RV on LV structure. Although our study showed no significant relationship between age-related data, with LV and RV GLS before and after PVR, there was an inverse meaningful correlation between patients’ age, age difference between rTOF and PVR and LVEF which reflects deterioration of LV function over time. Unexpectedly we obtained no significant correlation between RVEF and age related data, which might be supported by the positive effect of severe PR on RV stroke volume (Frank Starling law) (Table 2). As a matter of fact, there will be overestimated RV function and I wonder how much it is actually worsening over time.

Considering to RV function, there was a significant inverse correlation between RV GLS, RV Sm TAPSE and RV FAC, and also between LV GLS, LVEF and LVOT VTI which are in favor of agreement of conventional and deformation echocardiography together. There were also significant correlations between RV FAC & echo-derived LVEF and MRI-obtained RVEF & LVEF, respectively, which are all in favor of accuracy of echo measurements compared to MRI (Table 2); however, it is contrasted by the results of Burkhardt’s study in which neither LVEF, nor RVEF were changed significantly after PVR [23].

The reverse correlation between RV GLS and RV FAC was significant (*P* = 0.04), and there were also meaningful relationship between RV and LVGLS with LVEF and RVFAC, respectively, which later ones could be explained by ventricular interdependence (Table 2).

Patients with rToF have abnormal LV myocardial mechanics, as demonstrated by speckle-tracking echocardiography [9]; it should be noted that the negative impact of RV dilation on LV function has been linked to LV circumferential strain and strain rate (SR) in patients after TOF repair [20]. Many studies have been done to facilitate the optimal timing of PVR and to determine preop thresholds of RV volumes, above which no decrease or normalization of RV size takes place after surgery [24, 25].

Our study results are compatible with Yim’s study findings in 2017, in which mid-term post-PVR RV GLS decreased above pre-operative strain (− 19.2 ± 2.7 to − 22.0 ± 3.0%, *P* < 0.001) with declines observed in individual RV segments. LV and RV GLS were impaired early post-op, followed by recovery of biventricular systolic strain by mid-term follow-up [13]. Balasubramanian’s study also showed similar results to that of our study, at a mean of 7 months following PVR, in which LV global basal and apical strains and basal synchrony improved, but PVR had no impact on RV GCS and GLS [21]. The lack of improvement of RV functional indices has been probably recognized to be correlated with the fact that the restoration occurring after myocardial irreversible damage, so suggesting further studies with larger numbers whether earlier PVR leads to a better RV response. This is exactly what we followed in our patients, so better RV function might be expected (median 22y in their study, versus 12y in ours). In another study, PVR was also associated with improved LV and RV GLS in rTOF patients [19] which is in line with our study findings as well.

In contrast, in Harrington’s study in 2021, CMR-derived LV rotational mechanics in rTOF patients including rotation, twist, and torsion were diminished compared to control and did not improve at a median of 1 year after PVR despite favorable RV remodeling [26]. So was there in Beatrice Monti’s study, in which both global and segmental RV strains increased over time in patients undergone PVR as in patients who did not, and yearly variations did not differ significantly between groups [27]. These both could probably occur due to the older age group undergoing PVR compared to Yim’s and our study population, acknowledging in this study by the inverse correlation between age and LVEF, which might somehow be in favor of preservation of cardiac function with earlier intervention.

According to Masateru Kawakubo’s research, RV in patients with adapted remodeling is similar to the normal LV, which has a well-developed middle circumferential layer. Thus, the increase in the circumferential fiber mass may contribute to the predominant circumferential RV free-wall shortening, which supported by improved GCS (Global Circumferential Strain) and impaired GLS in the rTOF group relative to the control group. Furthermore, the researchers noted that even though the RV volume was reduced by PVR, there was no reverse remodeling of myocardial motion up to 1 year after the surgery. For that reason, earlier surgical intervention was suggested in order to prevent myocardial remodeling; what we exactly followed in our patients [28].

Among the contrasting studies to ours, is Pia Sjoberg et al. study which implied loss of atrioventricular coupling after PVR, possibly due to loss of pericardial integrity, denoting this complicates assessment of ventricular function after surgery using measurement of longitudinal function [29]. This incompatibility could somehow be explained by the different age limits (median 33y in their study, versus 12y in ours) which might account as an intervening factor, as approved by relative clinical improvement in general following this patients in more lengthy follow-ups.

Performing MRI, though well known as a better indicator for detection of accurate timing of PVR, is neither feasible, nor adequately accessible and nor cost effective. And it is sometimes impossible due to pacemakers, ICD, stents, coils and other impediments. Our study results, considering conventional and deformation echocardiography, could be a path forward in substituting echocardiography as a valuable handy modality, easy and safe to repeat, and accessible one; so holding promise to estimate the right time frame for performing PVR.

### Study limitation

In the present study, some limitation should be considered such as small sample size and short follow-up period that could be partly due to Covid-19 pandemic, decreasing the referral cases for elective surgery. Moreover, performing the study in pediatrics could be a rational reason, as timely PVR is less required in this age group. Longer follow-ups would be aimed in our next studies. Unfortunately, post-op. CMR could not be done during this study time period because of some drawbacks. We tried to overcome the issue by substituting echocardiographic measurements, LVEF by Simpson’s calculation and RVEF by RV FAC. Another note is that, respecting deformation echocardiography in the present study, be that as it may, lacks complete data, which can be explained by existence of some shortcomings of registering echocardiography system at the time of study. We would specially take that into consideration along with addition of some indices like GCS, torsion, twist, dyssynchrony and CMR principal strain in our relevant future studies.

## Conclusion

According to our study results, in those undergone PVR years following rTOF, using some echocardiographic indices, and based on the study protocol included a pre-op., an early and a late post-op. time sectors, a deduction could be emanate that the improving effects of surgical PVR were more evident after 6-month post-operation. Therefore, it supports potential long-term benefits of PVR. Moreover, it revealed an inverse correlation between patients’ age and LVEF, which might somehow be in favor of preservation of cardiac LV function with earlier intervention.

## Data Availability

All data are available upon request from the corresponding author.

## Abbreviations

rTOF: Repaired tetralogy of Fallot
PR: Pulmonary regurgitation
RV: Right ventricular
LV: Left ventricular
GLS: Global longitudinal strain
PVR: Pulmonary valvular replacement
2D: 2 Dimensional
Postop: Postoperation
MRI: Magnetic resonance imaging
CMR: Cardiac MRI
LVEF: LV ejection fraction
RVEF: RV ejection fraction
RV FAC: RV fraction area change
SD: Sudden death
CHB: Complete heart block
RVEDV: RV end-diastolic volume
RVESV: RV end-systolic volume
LV GCS: LV global circumferential strain
ICD: Intra cardiac defibrillator
VSD: Ventricular septal defect
LVOT VTI: LV outflow tract velocity time integral
TAPSE: Tricuspid annular plane systolic excursion
DTI: Doppler tissue imaging
SA: Surface area
RV Sm: Sprime=peak systolic velocity by tissue Doppler
